# Idiopathic remitting seronegative symmetrical synovitis with pitting edema syndrome with underlying history of malignancy

**DOI:** 10.1002/ccr3.7330

**Published:** 2023-05-09

**Authors:** Kain Kim, Charles Marvil, Bhavin B. Adhyaru

**Affiliations:** ^1^ Division of General Internal Medicine, Department of Medicine Emory University School of Medicine Atlanta Georgia United States

**Keywords:** arthritis, edema, polymyalgia rheumatica, RS3PE, seronegative arthritis, synovitis

## Abstract

**Key Clinical Message:**

By reporting this case, we hope to emphasize the importance of maintaining a high index of clinical suspicion for the early recognition of RS3PE in patients presenting with atypical symptoms of PMR and underlying history of malignancy.

**Abstract:**

Remitting seronegative symmetrical synovitis with pitting edema is a rare rheumatic syndrome of unknown etiology. It shares qualities with many other common rheumatological disorders such as rheumatoid arthritis and polymyalgia rheumatica, making diagnosis especially challenging. RS3PE has been speculated to be a paraneoplastic syndrome, and those cases associated with underlying malignancy have shown to respond poorly to conventional treatment. Therefore, it is advisable to routinely screen patients with malignancy and presenting with RS3PE for cancer recurrence, even if in remission.

## 1 INTRODUCTION

Remitting seronegative symmetrical synovitis with pitting edema (RS3PE) syndrome is an uncommon autoimmune disease in the seronegative arthritis category that presents with many similarities to polymyalgia rheumatica (PMR). First described by McCarty et al in 1985, it is characterized by acute symmetric tenosynovitis and pitting edema of dorsal hands and/or feet, affecting predominantly men and the elderly.^1^, ^2^ Inflammatory markers are usually elevated, with negative rheumatoid factor (RF) and anti‐citrullinated peptide (CCP) antibodies.^1^, ^2^, ^3^ The syndrome has a benign course and typically resolves with low‐dose glucocorticoids.^1^, ^2^, ^3^ RS3PE is often associated with other types of rheumatic diseases, as well as neoplastic conditions. When associated with underlying malignancy, the RS3PE syndrome is more often associated with solid tumors, particularly adenocarcinoma.^4^ In this report, we discuss a patient who was found to have RS3PE with a previous history of prostate cancer.

## 2 CASE PRESENTATION

A 62‐year‐old African American male with a history of prostate cancer presented to the emergency department (ED) with altered mental status after being found down in his apartment by his landlord. In the ED, the patient was minimally responsive, would not open his eyes, and was unable to move his extremities. Friends of the patient noted he was in his usual state of health the week prior but did have some difficulty with stepping into a car. Friends also noted that the patient had been complaining of shoulder pain, weakness, and difficulty completing tasks like dressing and bathing over the past couple months. The patient had received external beam radiation and hormone therapy 4 years prior; his last known and current PSA were negative. Further malignancy workup was not pursued at time of admission due to constraints of the inpatient setting.

On admission, vitals showed a temperature of 37.1°C, a heart rate of 138, respiratory rate of 20, blood pressure of 151/96, and an oxygen saturation of 100%. On examination, the patient was oriented to person only with a Glasgow Coma Scale score of E4V5M6. He appeared agitated and was diffusely tender to touch. He had dry oral mucosa, decreased skin turgor, ulcerated lesions on his lips and tongue, and a diffuse scaly, macular rash. There was also bilateral wrist swelling with pitting edema. He was unable to lift both his upper and lower extremities, had decreased range of motion in all extremities, and had generalized muscle atrophy more proximally than distally.

On admission, laboratory data showed elevated erythrocyte sedimentation rate (ESR) and C‐reactive protein of 86 mm/h and 8.34 mg/dL, respectively; his chemistry showed a Na 156 mmol/L, K of 5.2 mmol/L, BUN and Cr of 47 mg/dL and 1.6 mg/dL (unclear baseline). His creatine phosphokinase was normal, and his RF, CCP, antinuclear antibody, and rapid plasma reagin were all negative. His magnetic resonance imaging (MRI) was negative for stroke.

Initially, an inflammatory immune‐mediated cerebrovascular insult was suspected to be contributing to his altered mental status in light of the dermatological changes and elevated inflammatory markers. His rash was biopsied to rule out vasculitis or other rheumatological causes, and revealed epidermal spongiosis with eosinophilia. However, his altered mental status improved significantly with the administration of copious intravenous fluids. Aspiration of the left wrist joint was consistent with inflammatory arthritis, showing a lack of crystals with leukocytosis in the synovial fluid.

PMR was also considered due to the patient's muscle weakness and tenderness, tenosynovitis, and elevated inflammatory markers. Given his specific presentation, however, he was diagnosed with RS3PE syndrome and subsequently started on a low dose of prednisone (5 mg twice daily). The patient experienced dramatic improvement in strength afterward, resuming ambulation with a walker within a week. Yearly follow‐up has been conducted to present day with no relapses or recurrence of the symptoms.

## 3 DISCUSSION

Remitting seronegative symmetrical synovitis with pitting edema is a rare inflammatory arthritis with four main diagnostic criteria: localized edema on the dorsum of the hands/feet, acute onset of polyarthritis, age of at least 50 years, and seronegative for RF.^5^ Though our case satisfied all of these criteria, this patient additionally presented with less typical features like the ulcerated lesions and diffuse rash. Though evidence is lacking, a recent case report has observed additional skin manifestations like urticarial rash in a RS3PE patient.^6^

Our differential diagnosis included PMR, on which there has historically been some controversy on whether it constitutes the same syndrome as RS3PE. However, there are important distinctions between the two (Table 1). The pitting edema and peripheral arthritis of RS3PE is rare in PMR, which more classically presents with shoulder and pelvic girdle weakness, along with systemic signs. RS3PE is also more common in males, as opposed to PMR, which is more often seen in females. They are associated with different haplotypes, with RS3PE being associated with HLA‐B7, B27, and A2, while PMR is associated with HLA‐DR4. The conditions also differ in treatment, as RS3PE does respond to low‐dose steroids, NSAIDs, and sometimes hydroxychloroquine, while PMR only responds to steroids. Finally, RS3PE is associated with a better long‐term prognosis versus that of PMR, which has more frequent recurrences and relapses.^7^

**TABLE 1:** Characteristic differences between the RS3PE syndrome and PMR.

	RS3PE	PMR
Prevalence	More common in males Associated with underlying malignancy	More common in females
Joint involvement	Involves the wrist with pitting edema	Involves shoulder, pelvic girdle. Rarely has pitting edema
Genetics	HLA‐B7, B27, A2	HLA‐DR4
Treatment	Dramatic response to low‐dose steroids, NSAIDs, or hydroxychloroquine	Typically low‐dose steroids
Prognosis	Excellent long‐term prognosis	Frequent relapses and recurrences

Abbreviations: RS3PE, remitting seronegative symmetrical synovitis with pitting edema; PMR, polymyalgia rheumatica.

Late‐onset rheumatoid arthritis (LORA) was also considered, but this normally involves the large proximal, rather than small, joints.^8^ Crystal‐induced arthritis, such as gout or calcium pyrophosphate arthritis, can also mimic PMR but presents asymmetrically, with specific calcification findings on X‐ray or crystals on joint fluid aspiration.^9^

The RS3PE syndrome has been associated with underlying malignancies in up to 20% of cases, increasing to 30% for those coexisting with PMR.^4^ Most cases consist of solid tumors, the most common of which include prostatic, gastric, rectal, colic, endometrial, hepatocellular, pancreatic, and ovarian tumors. RS3PE can also be associated with various hematologic malignancies, including myelodysplastic syndrome, non‐Hodgkin's lymphoma, T‐cell lymphoma, and lymphocytic leukemia.^10^ Prior studies have suggested inflammatory processes of malignancy involving VEGF and TNF‐α may play a causative role in RS3PE, though the exact mechanism of pathogenesis has not yet been elucidated.^11^

Along with the characteristic features of RS3PE, the presence of other systemic signs like anorexia and weight loss could indicate an underlying paraneoplastic condition.^7^ For diagnostic markers, a multicenter retrospective study found that a high serum matrix metalloproteinase 3 (MMP‐3) level is characteristic of patients with paraneoplastic RS3PE syndrome.^12^ These patients may also require significantly higher doses of prednisone to show signs of improvement, unlike the rapid response to low‐dose corticosteroids seen in classic RS3PE patients. The best treatment of paraneoplastic RS3PE is to treat the malignant process itself.^13^ Though the patient in this case did have a history of prostate cancer, PSA levels were negative on admission and the patient showed no signs concerning for recurring malignancy, therefore less concerning for a directly paraneoplastic process; this is further evidenced by the resolution of his symptoms with low‐dose prednisone. Nevertheless, it is crucial to continually monitor for tumor recurrence, as RS3PE without apparent signs of malignancy at the time has been shown to precede cancer diagnosis for up to years at a time.^14^

## 4 CONCLUSION

Our case demonstrates that the clinical presentation of RS3PE syndrome can be misleading and easily mistaken for PMR. Those admitted for arthritis and edema should be considered for the diagnosis of RS3PE and include a workup for possible underlying malignancy. Early diagnosis of RS3PE can limit duration of corticosteroid therapy, which is crucial in elderly populations to avoid multiple side effects.

## AUTHOR CONTRIBUTIONS

**Kain Kim:** Writing – original draft; writing – review and editing. **Charles Marvil:** Conceptualization; project administration; writing – original draft. **Bhavin Adhyaru:** Conceptualization; project administration; supervision; writing – original draft; writing – review and editing.

## FUNDING INFORMATION

The authors received no financial support for the research, authorship, and/or publication of this article.

## CONFLICT OF INTEREST STATEMENT

The authors declare that there is no conflict of interest.

## CONSENT

Written informed consent was obtained from the patient to publish this report in accordance with the journal's patient consent policy.

## Data Availability

Data sharing not applicable to this article as no datasets were generated or analysed during the current study.
